# Latent profiles of childhood trauma in bipolar disorder: associations with objective and subjective cognitive functioning and quality of life

**DOI:** 10.3389/fpsyg.2026.1695797

**Published:** 2026-02-02

**Authors:** Ruoyun Ma, Lixia Zhong, Duoduo Lin, Zhiyi You, Xia Luo, Xiaoling Lin

**Affiliations:** 1 Department of Cardiology, China-Japan Friendship Hospital, Beijing, China; 2 School of Nursing, Sun Yat-sen University, Guangzhou, China; 3 The Eighth Affiliated Hospital, Sun Yat-sen University, Shenzhen, China; 4 Xiamen Xianyue Hospital, Xianyue Hospital Affiliated with Xiamen Medical College, Fujian Psychiatric Center, Fujian Clinical Research Center for Mental Disorders, Xiamen, China; 5 School of Nursing, Chongqing Medical University, Chongqing, China; 6 School of Nursing, Xiamen Medical College, Xiamen, China

**Keywords:** bipolar disorder, childhood trauma, cognition, latent profile, quality of life

## Abstract

**Introduction:**

Childhood trauma is associated with a more severe clinical course in bipolar disorders; however, the latent profiles of childhood trauma and their differential impacts on cognitive functioning and quality of life remain underexplored.

**Methods:**

Using latent profiles analysis, 275 bipolar patients were categorized into distinct trauma profiles based on Childhood Trauma Questionnaire scores. The characteristics of sociodemographic, clinical, pharmacological and biochemical variables, as well as objective and subjective cognitive functioning, and quality of life, were compared across profiles and with 63 healthy controls.

**Results:**

Three distinct profiles emerged: high trauma (*HT, 14.55%*), high neglect (*HN, 30.18%*) and low trauma (*LT, 55.27%*). Compared to healthy controls, all bipolar groups exhibited worse performance on nearly all aspects of objective and subjective cognitive functioning, and quality of life. Both the HT and HN profiles were significantly associated with subjective cognitive functioning. Notably, only the HT profile was significantly associated with objective cognitive functioning, whereas the HN profile was specifically linked to quality of life.

**Discussion:**

This study highlights multiple childhood trauma profiles in bipolar disorder. Findings reveal that trauma and neglect significantly influence diverse functional and clinical outcomes in bipolar disorder. Further research is crucial to elucidate their impact mechanisms.

## 1 Introduction

Childhood trauma, also referred to as adverse childhood experiences, encompasses all forms of physical or emotional abuse, sexual abuse, neglect, or other exploitation that occur in children or adolescents (Zhang et al., 2020), which has linked to poor developmental, behavioral, and functional outcomes (Dhondt et al., 2022; Fergusson et al., 2013; Yu et al., 2024). It is widespread, with global prevalence rates ranging from 13% for sexual abuse to 36% for emotional abuse (Stoltenborgh et al., 2015). A meta-analysis indicates that patients with bipolar disorder (*BD*) are 2.6 times more likely to have been exposed to childhood trauma compared with healthy individuals (Palmier-Claus et al., 2016). Furthermore, a history of childhood trauma is associated with a more severe clinical course, an increased risk for depressive episodes, rapid cycling, worse cognitive functioning, and a poorer quality of life (*QoL*) in patients with BD (Andrianarisoa et al., 2017; Buecker et al., 2013; Etain et al., 2013; Guillen-Burgos et al., 2023; Jiménez et al., 2017; Savitz et al., 2008).

While cognitive dysfunction in BD has been extensively studied (Elias et al., 2017; Torres et al., 2007), the role of childhood trauma—particularly its heterogeneous manifestations—in shaping both objective and subjective cognitive outcomes remains unclear (Buecker et al., 2013; Ehrlich et al., 2023; Jiménez et al., 2017; Marshall et al., 2016; Martins et al., 2019). Moreover, the mechanisms linking trauma subtypes to QoL in BD are yet to be elucidated. For example, Buecker et al.’s (2013) study showed that childhood trauma was associated with decreased auditory attention, verbal memory, and working memory among individuals with BD. Ehrlich et al.’s (2023) study showed that bipolar patients with higher childhood trauma performed worse visual and auditory memory than who with lower childhood trauma. These deficits correspond with the cognitive impairments observed in bipolar patients, indicating that childhood trauma could be an environmental contributory factor in the cognitive impairments and variability seen in BD (Ehrlich et al., 2023; Savitz et al., 2008). Self-reported cognitive difficulties in BD are potentially important, which are associated with neurocognitive functioning and mood symptoms based on our previous studies (Lin et al., 2019; Luo et al., 2020). Recent studies have found a dose–response relationship between childhood trauma and subjective cognitive functioning in American middle-aged and older adults (Baiden et al., 2022; Liu et al., 2024). Nonetheless, very few studies have focused on the relationship between childhood trauma and subjective cognitive functioning in individuals with mental disorders. Overall, to clarify the relationship between childhood trauma on both neurocognition and subjective cognition in BD is meaningful, as it could provide insight into an adverse experience that may have a far-reaching impact on an individual’s life.

Bipolar patients spend approximately half their life-time symptomatic symptoms, greatly affecting their QoL (Khafif et al., 2021). QoL is a comprehensive assessment of physical, emotional, and social/role functioning, serving as a critical outcome in evaluating disability and disease progression (Fayers and Machin, 2007). While QoL typically reflects facets of life that are most likely to be impacted health status changes, it is also associated with psychosocial status and may be influenced by distant environmental factors such as childhood trauma (Lin et al., 2018; Sun, 2021; Weber et al., 2016). However, little research has been conducted to determine the impact of childhood trauma on QoL in bipolar patients (Erten et al., 2014; Rowe et al., 2024; Serafini et al., 2016). Serafini et al. (2016) study revealed that childhood traumatic experiences may also be significantly correlated with extreme sensory processing patterns, which play an interesting role in the QoL of patients with affective disorders. Furthermore, bipolar patients with a history of childhood trauma had lower QoL and its subscale scores than those without (Erten et al., 2014). Nevertheless, it is essential to conduct further study to elucidate the impact of childhood trauma on the QoL in BD.

Exposure to a single traumatic event in childhood may increase the likelihood of experiencing additional traumatic events, suggesting that childhood traumas often co-occurred (Nilaweera et al., 2022). Person-centered modeling approaches, like latent profile analyses (*LPA*), has been used to empirically evaluate the co-occurrence of childhood trauma in individuals (Quinlan et al., 2020; Utzinger et al., 2016). For example, Utzinger et al.’s (2016) study identified four profiles: low/no trauma, emotional trauma, sexual trauma, and polytrauma, with sexual or polytrauma groups showing higher psychopathology levels linked to borderline personality disorder. LPA, an extension of latent class analysis for continuous variables, identifies subgroups with distinct trauma patterns (Vermunt and Magidson, 2005). Understanding risk characteristics associated with these profiles can aid in designing personalized clinical interventions.

Research on latent profiles of childhood trauma in BD samples is nearly nonexistent. Thus, the current study aims to: i) identify childhood trauma profiles in BD using the LPA; ii) compare various profiles characteristics across sociodemographic, clinical, pharmacological and biochemical variables, and objective and subjective cognitive functioning, as well as QoL; and iii) explore the predictive effects of trauma profiles on objective and subjective cognitive functioning and QoL. We hypothesize that: i) latent profiles of childhood trauma can be identified in BD; ii) patients in different trauma profiles will exhibit varying cognitive functioning and QoL outcomes; and iii) childhood trauma profiles will predict both objective and subjective cognitive functioning, as well as QoL.

## 2 Methods

### 2.1 Participants

The present study employed a cross-sectional design. Individuals with BD were recruited at the outpatient psychiatric units of three tertiary hospitals located in Guangzhou and Xiamen, China, from April 2019 to April 2021. Inclusive criteria for bipolar patients were: i) a diagnosis of BD confirmed by two psychiatrists using the Structured Clinical Interview for DSM-V Axis I Disorders, Clinical version (First et al., 2016), combined with the 17-item Hamilton Depression Rating Scale (*HDRS-17*) (Hamilton, 1960) and the Young Mania Rating Scale (*YMRS*) (Young et al., 1978); ii) 16–60 years old; iii) had received a level of junior high school education or above. Exclusive criteria included: i) presence of severe depressive or manic episodes or current active psychotic symptoms; ii) had severe physical or neurological illness; iii) with a history of head injury; iv) intellectual disability (Wechsler Adult Intelligence Scale score < 70); v) drug abuse or alcohol dependence within the last year; and vi) receipt of electroconvulsive therapy within the past 6 months. Healthy individuals (*HC*) matched by age, gender and educational level were recruited using a convenience sampling method from two communities in Guangzhou, China. Exclusive criteria for HC were: i) a diagnosis of any psychiatric diseases; and ii) having a first-degree relative with psychiatric diseases. Finally, a total of 275 BD participants aged 28.44 (*SD = 9.88, 34.90% male*) and 63 healthy individuals aged 30.79 (*SD = 8.77, 34.92% male*) were included. All participants provided written informed consent prior to participation. The study received ethical approval from the Ethics Committee of Sun Yat-sen University.

### 2.2 Assessments

#### 2.2.1 Sociodemographic, clinical, and pharmacological variables

Sociodemographic variables (including age, gender, educational level, marriage, employment, residence, body mass index, waist-to-hip ratio, central obesity), clinical characteristics (including depressive and hypo/manic symptoms, age at onset, duration of illness, number of hospitalizations, and number of total, hypo/manic, depressive, mixed episodes, family psychotic history, history of physical illness, previous psychotic symptoms), and pharmacological treatments were collected via structured interviews and medical records. Please see Table 1.

**Table 1:** Descriptive summary and group differences of demographic and clinical characteristics between patients with bipolar disorder and healthy individuals.

Variables	High trauma (*N* = 40)	High neglect (*N* = 83)	Low trauma (*N* = 152)	Healthy controls (*N* = 63)	MANOVA	
Sociodemographic variables	Mean ± SD/*N* (%)				*χ*^2^/*F*	*p*	Significantdifference
Age (years)	29.83 ± 9.98	27.60 ± 10.03	28.54 ± 9.79	30.79 ± 8.77	1.45	0.22	
Gender (male)	15 (37.50)	25 (30.12)	56 (34.92)	22 (34.92)	1.21	0.75	
Educational level (years)	11.19 ± 2.91	10.88 ± 3.17	12.45 ± 2.88	12.57 ± 2.80	−1.85	0.07	
Being single (yes)	27 (76.50)	64 (77.11)	107 (70.39)	40 (63.49)	3.40	0.33	
Being on the job (yes)	8 (20.00)^a^	15 (18.07)^a^	34 (22.37)^a^	52 (80.56)^b^	90.10	<0.001	[HT, HN, LT] < HC**
Residence					26.02	<0.001	
Live alone	2 (5.00)^a,b^	5 (6.02)^a,b^	6 (3.95)^b^	10 (15.87)^a^			LT < HC**
Live with families	14 (35.00)^a^	33 (39.76)^a^	68 (44.74)^a,b^	39 (61.90)^b^			HT < HC**, HN < HC**
Live with others	24 (60.00)^a^	45 (54.22)^a^	78 (51.32)^a^	14 (22.22)^b^			[HT, HN, LT] > HC**
Body Mass Index (Kg/m^2^)	22.49 ± 5.48	22.15 ± 4.82	22.67 ± 3.85	22.82 ± 4.64	0.34	0.80	
Waist-to-Hip Ratio	0.88 ± 0.10	0.85 ± 0.09	0.87 ± 0.10	0.83 ± 0.07	0.94	0.42	
Central obesity (yes)	24 (60.00)^a^	40 (48.20)^a,b^	76 (50.00)^a,b^	20 (31.70)^b^	9.17	0.03	HT > HC**
Clinical Variables							
The HDRS score	8.48 ± 8.04	10.90 ± 7.72	7.95 ± 7.15	1.67 ± 1.80	23.04	<0.001	[HT, HN, LT]>HC***; HN>LT**
The YMRS score	6.25 ± 5.67	6.49 ± 6.12	6.43 ± 5.65	0.62 ± 1.20	20.83	<0.001	[HT, HN, LT]>HC***
Age at onset (years)	22.33 ± 8.30	19.76 ± 6.95	21.42 ± 7.56	–	1.99	0.14	
Duration of illness (years)	7.97 ± 8.05	7.51 ± 7.82	6.92 ± 6.94	–	0.30	0.74	
Number of hospitalizations	6.08 ± 8.19	3.36 ± 4.45	3.13 ± 4.08	–	5.25	0.006	HT>HN*; HN>LT**
Number of total episodes	8.92 ± 8.72	6.28 ± 4.88	5.37 ± 4.91	–	5.91	0.003	HT>LT**
Number of hypo/manic episodes	5.49 ± 5.53	3.49 ± 4.48	3.31 ± 4.11	–	3.50	0.03	HT>LT*
Number of depressive episodes	2.80 ± 5.66	1.83 ± 1.91	1.45 ± 1.92	–	3.80	0.02	HT>LT*
Number of mixed episodes	0.62 ± 1.12	0.95 ± 1.31	0.61 ± 0.96	–	2.93	0.06	
Family psychotic history (yes)	10 (25.00)	18 (21.69)	31 (20.39)	–	0.40	0.82	
History of physical illness (yes)	6 (15.00)	8 (9.64)	18 (11.84)	–	0.77	0.68	
Previous psychotic symptoms (yes)	19 (47.50)	28 (33.73)	56 (36.84)	–	2.24	0.33	
Medication							
Lithium	20 (50.00)	27 (32.53)	62 (40.79)	–	3.63	0.16	
Valproate	21 (52.50)	38 (45.78)	67 (44.08)	–	0.91	0.64	
Lamotrigine	1 (2.50)	8 (9.64)	6 (3.95)	–	4.16	0.13	
Oxcarbazepine	0 (0.00)	7 (8.43)	15 (9.87)	–	4.22	0.12	
Antipsychotics	36 (90.00)	66 (79.52)	122 (80.26)	–	2.28	0.32	
Antidepressants	0 (0.00)	6 (7.23)	6 (3.98)	–	3.52	0.17	
Benzodiazepines	8 (20.00)	28 (33.73)	38 (25.00)	–	3.22	0.20	
Benzhexol	4 (10.00)^a,b^	15 (18.07)^a^	8 (5.26)^b^	–	9.95	0.007	HN > LT**
Propranolol	1 (2.50)	5 (6.02)	12 (7.89)	–	1.56	0.46	
Serum lipid levels							
HDL (mmol/L)	1.28 ± 0.05	1.31 ± 0.10	1.26 ± 0.03	–	0.19	0.83	
LDL (mmol/L)	2.53 ± 0.11	2.56 ± 0.07	2.64 ± 0.05	–	0.58	0.56	
TG (mmol/L)	1.13 ± 0.09	1.30 ± 0.07	1.34 ± 0.07	–	1.10	0.34	
TC (mmol/L)	4.34 ± 0.14	4.21 ± 0.08	4.76 ± 0.40	–	0.69	0.50	
APoA1 (g/L)	1.22 ± 0.03	1.27 ± 0.02	1.34 ± 0.08	–	0.46	0.63	
APoB (g/L)	0.76 ± 0.05	0.76 ± 0.02	0.77 ± 0.02	–	0.45	0.64	

*p < 0.05, *p* < 0.01**, *p* < 0.001***. Central obesity, WHR ≥ 0.90 (male) or WHR ≥ 0.85 (female); HDRS, Hamilton Depression Scale; YMRS, Young Mania Rating Scale; HDL, high-density lipoprotein; LDL, low-density lipoprotein; TG, triglyceride; TC, total cholesterol; APoA1, apolipoprotein A-I; APoB, apolipoprotein B; HT, high trauma; HN, high neglect; LT, low trauma. a, b, If there were same marked letters between two groups, the difference between them was not statistically significant.

#### 2.2.2 Childhood trauma

The Childhood Trauma Questionnaire (*CTQ*) is a valid self-reported 28-item scale used to evaluate five types of childhood trauma, including emotional abuse, physical abuse, sexual abuse, emotional neglect, and physical neglect (Bernstein et al., 2003). Each item rated on a 5-point scale from 1 (*never true*) to 5 (*very often true*), with three items dedicated to validity assessment. Counter-scoring was required on item 2, 5, 7, 13, 19, 26, and 28. Each subscale ranged from 5 to 25 scores, and the total score ranged from 25 to 125, with higher scores indicating greater degree of childhood trauma. The Cronbach’s *α* of the CTQ was 0.81 among Chinese samples (He et al., 2019).

#### 2.2.3 Objective cognitive functioning

Patients with BD were tested with a comprehensive array of neuropsychological assessments exploring four cognitive domains: attention and processing speed, working memory, visual memory, and executive function. For a more detailed description of the neuropsychological assessments, please see Lin et al. (2020).

#### 2.2.4 Subjective cognitive functioning

The Cognitive Complaints in Bipolar Disorder Rating Assessment (*COBRA*), a unidimensional self-report scale, was used to assess the subjective cognition in bipolar patients. The COBRA consists of 16 items, each rated on a 4-point scale ranging from 0 (*never*) to 4 (*always*), where higher scores indicating greater impairments (Rosa et al., 2013). The Chinese version of the COBRA has been validated and demonstrated to be a reliable instrument for assessing cognitive impairments in Chinese patients with BD (Xiao et al., 2015).

#### 2.2.5 Quality of life

The Short Form 12-Item Health Survey (*SF-12*) is a widely used self-report instrument to measure QoL (Ware et al., 1995). It contains 12 questions and 8 subscales: general health, physical functioning, role-physical, bodily pain, vitality, social functioning, role-emotional, and mental health. The standard Physical Component Summary (*PCS*) and Mental Component Summary (*MCS*) scores were calculated by the standard algorithm described in the SF-12 Manual, with higher scores denoting better physical and health mental health (Ware et al., 1995). The Cronbach’s *α* value (*0.91*) reflected satisfactory reliability of SF-12 among Chinese sample (Lam et al., 2005).

#### 2.2.6 Serum lipid levels

A lipid profiles included triglycerides (*TG*), total cholesterol (*TC*), low density lipoprotein cholesterol (*LDL-C*), high density lipoprotein cholesterol (*HDL-C*), apolipoprotein A1 (*ApoA1*), and apolipoprotein B (*ApoB*) were collected based on previous investigations (Godin et al., 2021). And the blood samples were placed in a blood collection tube with special measuring instruments by professionals in these two hospitals. The biochemical analyses were performed by technician who was blind to the clinical conditions of the patients, using and automatic biochemical analysis system.

### 2.3 Data analyses

Firstly, 275 patients with BD were selected for the LPA using Mplus version 7.4. The latent profile indicators encompassed the five subscales of the CTQ. In conducting the LPA, models were estimated using maximum likelihood estimation. Then, a series of models were sequentially fitted, ranging from a 1-profile to a 5-profile model. Model fit parameters included the Akaike information criterion (*AIC*), the Bayesian information criterion (*BIC*), sample size adjusted BIC (*aBIC*), bootstrapped likelihood ratio test (*BLRT*), Lo–Mendell–Rubin (*LMR*), and entropy. The AIC, BIC, and aBIC were used to compare the goodness-of-fit among competing models, with smaller values indicating a better model fit (Vermunt and Magidson, 2005). The BLRT and LMR tests assessed the difference in fit between the *k*-1 and *k*-profile models, with their *p*-values indicating statistically significant improvements in model fit for the estimated model over the *k*-1-profile model (Nylund et al., 2007). Entropy, which evaluates classification accuracy, is considered adequate when it exceeds 0.80 (Carragher et al., 2009). Other considerations encompass a sufficient sample size, high posterior classification probabilities for the individual latent profile, and the clinical significance of the identified profiles (Wang et al., 2023).

Secondly, SPSS version 25.0 was used for descriptive comparison and linear regression analyses. The categorical variables were presented as frequencies (*N*) and percentage (*%*). For continuous variables, normality was tested using histograms and P–P plots. Means and standard deviations (*SD*) were calculated. The four groups (*high trauma, high neglect, low trauma and HC*) were compared regarding sociodemographic, clinical, pharmacological and biochemical characteristics, the CTQ, objective/subjective cognitive functioning and QoL by using the chi-square tests (*χ^2^*) and one-way analysis of variance (*ANOVA*), as appropriate. Independent samples *t*-tests or *Turkey’s post-hoc* comparisons were performed to explore the differences between two groups (*any two of four groups*). Raw score from all neuropsychological tests were standardized to *z*-scores using the formula *z = (x - μ) / σ*, where *μ* and *σ* represent the mean and standard deviation of scores from HC. To ensure unidirectionality across all measures, *z*-scores derived from the COBRA and the Trail Making Test Part A and B, where higher scores denote poorer performance, were inverted. Composite scores for each of the four cognitive domains were calculated by averaging the relevant *z*-scores. A global cognitive functioning score was then computed as the mean of these domain *z*-scores, with lower scores indicating worse function. Score of SF-12, PCS, and MCS were also standardized to *z*-scores based on HC.

Lastly, hierarchical linear regression analyses examined whether childhood trauma profiles could predict cognitive functioning and QoL in bipolar patients. The global and four cognitive domains’ scores, the COBRA score, and the SF-12 score were treated as dependent variables in separated models. As for independent variables, several potential confounders as covariates were entered in the first block, including the HDRS and YMRS scores. In the second block, several variables were selected based on the prior results and previous studies (Buecker et al., 2013; Ehrlich et al., 2023; Lin et al., 2020), including childhood trauma profiles, years of education, work status, and duration of illness. All independent variables were entered using a stepwise method. All tests were two-tailed and statistical significance was defined as a value of *p* < 0.05.

## 3 Results

### 3.1 Profiles of childhood trauma

**Figure 1:**
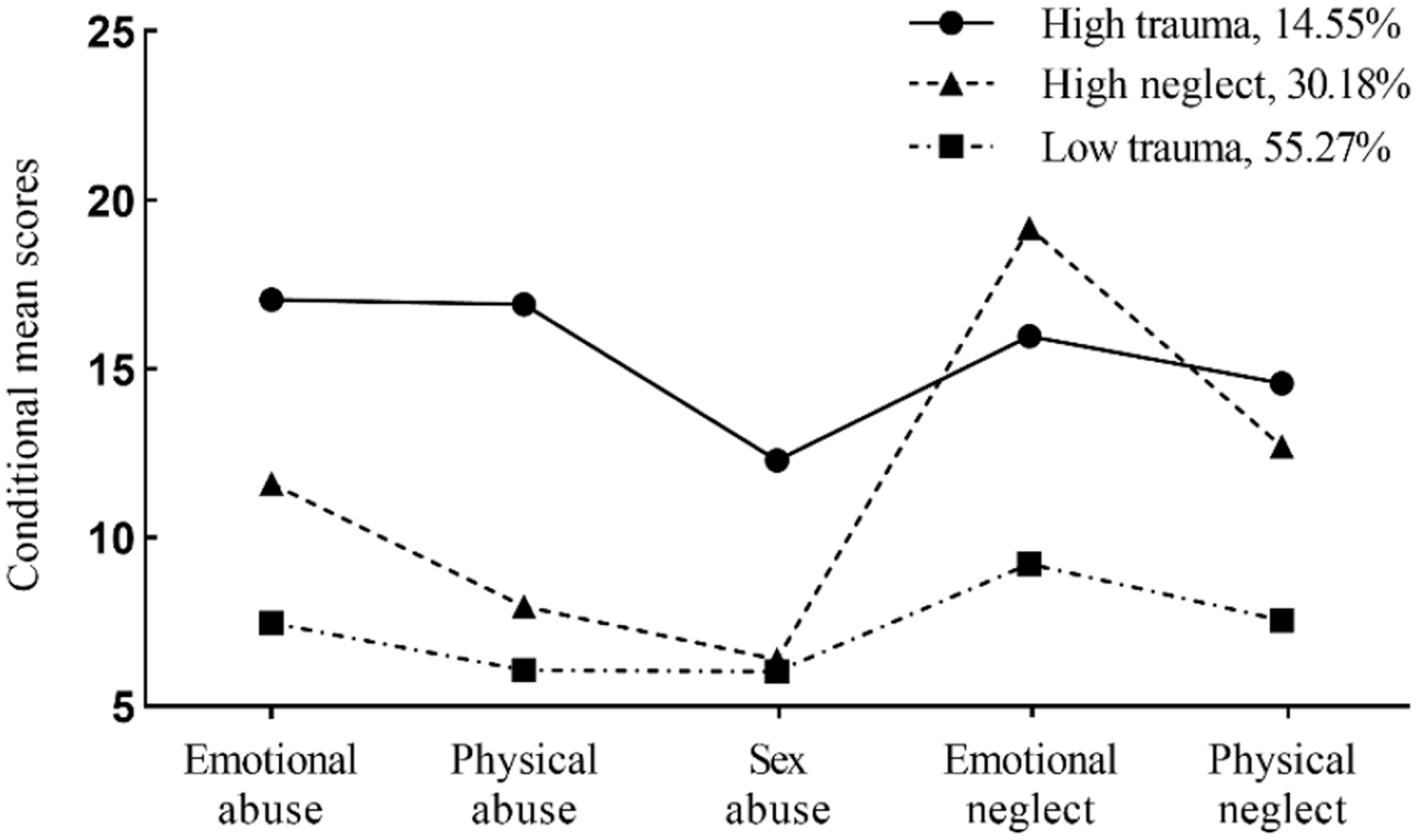
Mean scores of childhood trauma by classes of three-profile model (*N* = 275).

As shown in Table 2 and Figure 1, the 3-profile-model was optimal for evaluating childhood trauma in bipolar patients. AIC, BIC, and aBIC decreased, suggesting an improvement in the model. The 4-profile model’ s LMR was not significant (*p* = 0.13), whereas both the 3-profile-model’s LMR and BLRT *p-*values were below 0.05, indicating a better fit. The entropy values above 0.80 for the 3-profile-model suggested good classification accuracy. Latent profile probabilities of profile 1, 2, and 3 were 95.8, 90.5, and 96.9%, respectively, indicating good LPA reliability. Profile 1 (*N* = 40, 14.55%) grouped patients experiencing high levels of all types of traumas, was labeled ‘High Trauma’ (*HT*). Profile 2 (*N* = 83, 33.18%) characterized by high levels of emotional neglect and physical neglect, was thus termed ‘High neglect’ (*HN*). Profile 3 (*N* = 152, 55.27%), representing minimal trauma across all five types, was termed ‘Low trauma’ (*LT*).

**Table 2:** Fit indices for five models using latent profile analysis (*N* = 275).

Profile	AIC	BIC	aBIC	LMR *p*-value	BLRT *p*-value	Entropy	Group size for each profile				
							1	2	3	4	5
1	8103.59	8139.76	8108.05	–	–	–	–	–	–	–	–
2	7726.30	7784.17	7733.44	0.02	<0.001	0.89	206	69	–	–	–
**3**	7553.63	7633.20	7563.44	0.01	<0.001	0.88	152	83	40	–	–
4	7454.20	7555.47	7466.68	0.13	<0.001	0.91	152	79	30	14	–
5	7367.20	7490.17	7382.37	0.06	<0.001	0.93	151	73	33	14	4

AIC, Akaike information criterion; BIC, Bayesian information criterion; aBIC, adjusted Bayesian information criterion; LMR, Lo–Mendell–Rubin; BLRT, bootstrapped likelihood ratio tests; Bold type indicates the selected best fit model.

### 3.2 Group differences of sociodemographic, clinical, pharmacological and biochemical characteristics across four groups

As shown in Table 1, there were no significant differences on demographic data among the BD and HC groups (all *p* > 0.05), except for working status, residence and central obesity (*p* < 0.01). Regarding the clinical variables, the three BD groups showed significantly higher scores on the HDRS-17 and the YMRS compared to the HC group. Furthermore, no significant differences were observed in clinical, pharmacological and serum lipid variables among three BD groups (all *p* > 0.05), except that the HT group had higher number of hospitalizations and episodes (total, hypo/manic and depressive) than the LT group (*p* < 0.05), and the HN group patients used benzhexol more frequently than the LT group (*p* < 0.05).

### 3.3 Associations between latent profiles of childhood trauma and objective/subjective cognitive functioning and quality of life

**Figure 2:**
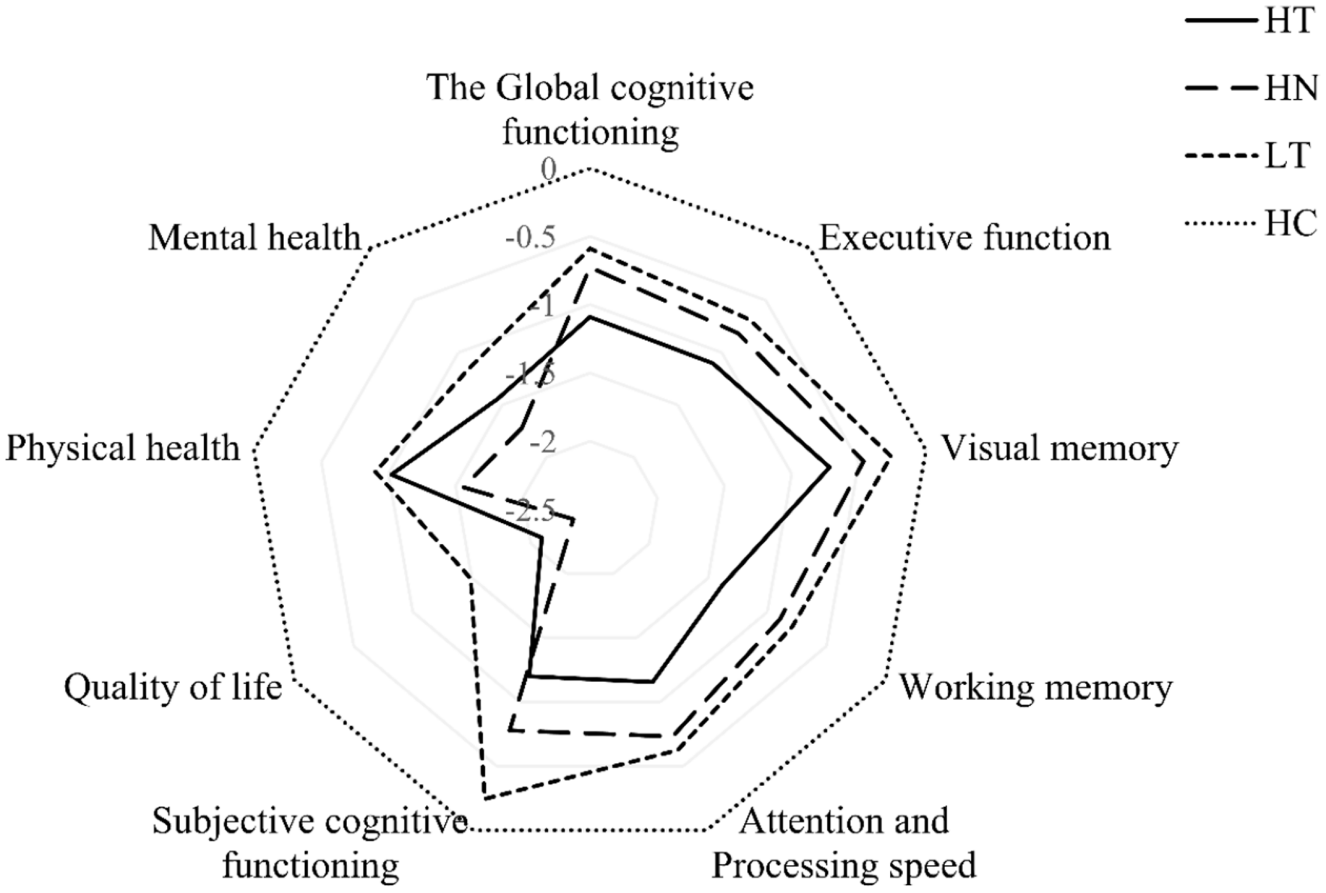
Radar plot depicting group differences in objective and subjective cognitive functioning, and quality of life. HT, high trauma; HN, high neglect; LT, low trauma; HC, healthy control.

As shown in Table 3 and Figure 2, there were significant differences in objective and subjective cognitive functioning, and quality of life across the four groups. All BD profiles (*HT, HN and LT*) demonstrated significant impairments in global cognitive functioning (attention, working memory and executive function) and QoL compared to HC (all *p* < 0.05). Figure 2 provides a graphical depiction of group differences across these four groups. The HT group performed poorer global and domain-specific cognitive functioning than the LT group (*p* < 0.05), except for visual memory (*p* > 0.05). Both the HT (*p* < 0.001) and HN (*p* = 0.001) groups had worse subjective cognitive functioning than the LT group. The HN group also scored lower in QoL and its two subscales (*p* < 0.05) compared to the LT group. No significant differences were found between the HT and HN groups, or the HN and LT groups in objective cognitive functioning (*p* > 0.05).

**Table 3:** Childhood trauma, objective and subjective cognitive functioning, and quality of life across four groups.

Variables	High trauma (*N* = 40)	High neglect (*N* = 83)	Low trauma (*N* = 152)	Healthy controls (*N* = 72)	ANOVA		Turkey *Post-hoc* tests
	Mean ± SD				*F*	*p*	
The Childhood Trauma Questionnaire score	77.10 *±* 10.55	57.86 *±* 8.12	36.16 *±* 7.30	36.49 *±* 10.46	320.33	<0.001	HT > HN > LT^***^; [HT, HN] > HC^***^
Emotional abuse	17.05 *±* 4.95	11.61 *±* 4.70	7.45 *±* 2.71	7.16 *±* 2.63	93.95	<0.001	HT > HN > LT^***^; [HT, HN] > HC^***^
Physical abuse	16.95 *±* 3.76	7.94 *±* 2.96	6.07 *±* 2.03	5.86 *±* 1.67	218.18	<0.001	HT > HN > LT^***^; [HT, HN] > HC^***^
Sexual abuse	12.45 *±* 5.38	6.29 *±* 2.09	6.06 *±* 2.37	5.32 *±* 0.95	70.61	<0.001	HT > [HN, LT] ^***^; HT > HC^***^
Emotional neglect	16.03 *±* 5.12	19.20 *±* 4.05	9.12 *±* 3.19	10.14 *±* 5.04	128.44	<0.001	HT > HN > LT^***^; [HT, HN] > HC^***^
Physical neglect	14.63 *±* 2.85	12.81 *±* 2.70	7.45 *±* 2.56	8.02 *±* 3.12	120.75	<0.001	HT > HN^**^, [HT, HN] > LT^***^; [HT, HN] > HC ^***^
The Global Cognitive Functioning^a^	−1.09 *±* 0.99	−0.73 *±* 1.07	−0.59 *±* 0.89	≈0.000001 *±* 0.61	13.39	<0.001	HT < LT ^*^; [HT, HN, LT] < HC^***^
Attention and Processing speed^a^	−1.16 *±* 1.28	−0.73 *±* 1.26	−0.62 *±* 1.05	≈0.000001 *±* 0.73	10.34	<0.001	HT < LT^*^; [HT, HN] < HC^***^, LT < HC^**^
Working memory^a^	−1.38 *±* 1.26	−0.89 *±* 1.44	−0.79 *±* 1.33	≈0.000001 *±* 0.80	11.05	<0.001	HT < LT ^*^; [HT, HN, LT] < HC^***^
Visual memory^a^	−0.72 *±* 1.35	−0.47 *±* 1.45	−0.26 *±* 1.15	≈0.000001 *±* 0.85	3.44	0.017	HT < HC^*^
Executive function^a^	−1.10 *±* 1.14	−0.81 *±* 0.92	−0.68 *±* 0.77	≈0.000001 *±* 0.71	17.24	<0.001	HT < LT^*^; [HT, HN, LT] < HC^***^
Subjective Cognitive Functioning^b^	−1.76 *±* 1.99	−1.14 *±* 1.70	−0.36 *±* 1.49	≈ − 0.000006 *±* 0.999	15.45	<0.001	HT < LT ^***^, HN < LT ^**^; [HT, HN] < HC^***^
Quality of Life^c^	−2.09 *±* 1.81	−2.36 *±* 1.71	−1.49 *±* 1.76	≈0.000778 *±* 0.999	26.96	<0.001	HN < LT^*^; [HT, HN, LT] < HC^***^
Physical health^d^	−1.02 *±* 1.53	−1.56 *±* 1.71	−0.90 *±* 1.53	≈0.000576 *±* 1.00	13.06	<0.001	HN < LT^**^; HT < HC^**^, [HN, LT] < HC^***^
Mental health^e^	−1.44 *±* 1.50	−1.72 *±* 1.44	−1.14 *±* 1.53	≈0.000502 *±* 1.00	18.80	<0.001	HN < LT^*^; [HT, HN, LT] < HC^***^

HT, high trauma; HN, high neglect; LT, low trauma; HC, healthy control. *p < 0.05, *p* < 0.01**, *p* < 0.001***. a, *z*-scores of the administered tests based on HC; b, *z*-score of the Cognitive Complaints in Bipolar Disorder Rating Assessment based on HC; c, *z*-score of the 12-item Short Form Health Survey based on HC; d, *z*-score of the Physical Component Summary based on HC; e, *z*-score of the Mental Component Summary based on HC.

Table 4 showed that the HT profile was significantly associated with all global and domain-specific cognitive functioning in patients with BD (*p* < 0.05), except for visual memory (*p* > 0.05). Both the HT (*p* < 0.001) and HN (*p* < 0.05) profiles was significantly associated with subjective cognitive functioning. Furthermore, only the HN profile was significantly associated with poorer quality of life (*p* < 0.05).

**Table 4:** Predicting effects of childhood trauma on objective and subjective cognitive functioning, and quality of life.

Variables	Attention and processing speed		Working memory		Visual memory		Executive functioning		Global cognitive functioning		Subjective cognitive functioning		Quality of life		Physical health		Mental health	
	*β*	*p*	*β*	*p*	*β*	*p*	*β*	*p*	*β*	*p*	*β*	*p*	*β*	*p*	*β*	*p*	*β*	*p*
Age	−0.43	<0.001	−0.34	<0.001	−0.37	<0.001	−0.41	<0.001	−0.46	<0.001	0.22	0.004	0.13	0.08	−0.07	0.39	0.21	0.005
Education	0.22	<0.001	0.21	<0.001	0.25	<0.001	0.24	<0.001	0.27	<0.001	−0.06	0.27	−0.11	0.04	−0.10	0.10	−0.12	0.03
Being on the job	0.06	0.28	0.01	0.83	0.05	0.38	0.04	0.48	0.04	0.45	−0.09	0.11	−0.02	0.67	−0.05	0.37	−0.01	0.89
Duration of illness	−0.01	0.91	−0.06	0.42	0.03	0.68	0.10	0.18	0.01	0.87	−0.17	0.02	−0.04	0.52	−0.02	0.75	−0.05	0.44
HT	−0.11	0.04	−0.11	0.04	−0.08	0.16	−0.12	0.04	−0.12	0.02	−0.29	<0.001	−0.08	0.12	−0.02	0.71	−0.09	0.11
HN	−0.02	0.72	−0.04	0.53	−0.06	0.29	−0.03	0.63	−0.05	0.40	−0.13	0.02	−0.14	0.01	−0.12	0.04	−0.11	0.04
LT	Ref		Ref		Ref		Ref		Ref		Ref		Ref		Ref		Ref	
HDRS score	0.04	0.52	0.20	0.001	0.15	0.02	−0.03	0.69	0.13	0.03	−0.41	<0.001	−0.57	<0.001	−0.49	<0.001	−0.47	<0.001
YMRS score	0.06	0.35	0.04	0.56	−0.02	0.72	−0.02	0.78	0.02	0.76	0.02	0.79	0.13	0.03	0.24	<0.001	0.003	0.99
Goodness of fit of the model																		
*∆R^2^*	0.226		0.188		0.180		0.174		0.271		0.110		0.037		0.023		0.047	
*F*	14.21		15.74		11.19		7.54		19.52		15.56		18.74		9.13		17.43	
*p*	<0.001		<0.001		<0.001		<0.001		<0.001		<0.001		<0.001		<0.001		<0.001	
VIF (highest)	2.25		2.25		2.25		2.25		2.25		2.25		2.25		2.25		2.25	

HT, high trauma; HN, high neglect; LT, low trauma; HDRS, Hamilton Depression Scale; YMRS, Young Mania Rating Scale; Ref, reference; VIF, variance inflation factor.

## 4 Discussion

In the study, we identified three childhood trauma profiles in bipolar patients: high trauma (*HT, 14.55%*), high neglect (*HN, 30.18%*) and low trauma (*LT, 55.27%*). Compared to HC, three BD groups (*HT, HN, LT*) exhibited worse performance across nearly all aspects of objective (attention and processing speed, working memory and executive function) and subjective cognitive functioning, as well as QoL. Notably, the HT group exhibited poorer global and domain-specific cognitive functioning compared to the LT group, except for visual memory. Both the HT and HN groups performed worse on subjective cognitive functioning compared to the LT group, and the HN group reported a lower QoL. After controlling for depressive and manic symptoms, the HT profile had a trend for lower objective cognitive functioning in three out of four domains compared with LT group. Furthermore, both the HT and HN profiles were significantly associated with lower subjective cognitive functioning, whereas only the HN profile was specifically linked to quality of life.

Three childhood trauma profiles were identified in our study, aligning with a recent study (Wang et al., 2023): high childhood trauma with high levels of all trauma types (*9.3%*), moderate trauma with emotional abuse and childhood neglect (*28.9%*), and no or low traumas (*61.8%*). Notably, 33.18% of bipolar patients belonged to the HN group in this study, marked by significant childhood neglect and moderate emotional abuse, highlighting the co-occurrence in affective disorders. Despite increasing research on childhood trauma subtypes, emotional and physical neglect remain understudied and often overlooked (Carvalho et al., 2024). Neglect has invisible yet long-lasting consequences, increasing the risk of mood disorder. In our study, 14.55% of bipolar patients were in the HT group, slightly higher than depressive patients in the recent study (Wang et al., 2023). Etain et al.’s (2010) study also noted a high prevalence of polytrauma in bipolar patients. However, Xie et al. (2018) study found that neither BD nor depression patients reported more than four childhood trauma types. These discrepancies may stem from methodological differences, particularly in categorizing trauma from continuous to categorical variables using varying cutoff points.

Consistent with previous studies (Ehrlich et al., 2023; Hsueh et al., 2024; Jiménez et al., 2017; Marshall et al., 2016; Miskowiak et al., 2023), our data also reveal that childhood trauma is associated with impaired cognitive functioning in BD, particularly in attention and processing speed, working memory, and executive function. Our study offers new sights into the role of latent profiles of childhood trauma, indicating that a high trauma profile might be a risk factor of objective cognitive dysfunctions. This aligns with the dose–response effect of later-life cognitive functioning and dementia risk (Nilaweera et al., 2022). Buecker et al.’s (2013) have demonstrated that childhood trauma was related to neurocognitive functioning in patients recently recovered from their first manic episode, longitudinal studies are needed to explore whether this relationship intensifies with disease progression. Hsueh et al.’s (2024) used a person-centered modeling approach, clustering 55 bipolar patients into low trauma, neglect-focused, and multiple-trauma groups. And only the neglect-focused group showed negative impacts on working memory compared to the low trauma group, potentially due to the small sample size limiting cluster analysis effectiveness. Previous study found that bipolar patients with high childhood trauma levels performed poorly on visual memory tasks, a finding not replicated in our study (Ehrlich et al., 2023). They dichotomized trauma levels using a cut-off one standard deviation above the mean CTQ score of healthy individuals. These differing results suggest that the impact of childhood trauma on cognitive functioning in BD is influenced not only by the cumulative effect of traumatic events but also by specific profile characteristics. Childhood trauma may impair objective cognitive functioning in BD through mechanisms involving elevated cortisol, C-reactive protein levels, and genetic factors like the low-activity Met allele of the brain-derived neurotrophic factor gene and the epsilon 4 allele of apolipoprotein E gene (Aas et al., 2019; Congio et al., 2022; Savitz et al., 2007). Magnetic resonance imaging studies indicate that gray matter volume alterations underlie cognitive disturbances in trauma-exposed BD patients (Begemann et al., 2023; Hsieh et al., 2021; Jørgensen et al., 2023). Despite cognitive impairment being a crucial treatment target in BD, effective evidence-based pro-cognitive treatments are limited (Miskowiak et al., 2022). Future research should explore how childhood trauma-induced neurobiological abnormalities contribute to BD and affect specific cognitive domains.

As expected, our findings aligned with prior research (Baiden et al., 2022; Liu et al., 2024) showing that childhood trauma was linked to poor subjective cognitive functioning in community adult populations, including those with BD. Although previous studies reported a dose–response relationship, our analysis found no significant differences in subjective cognitive functioning between HT and HN groups. This discrepancy may stem from our use of a 16-item self-reported measure, contrasting with the binary variable based on a single question used in earlier studies. Notably, subjective cognitive complaints during psychiatric assessment may signal a history of childhood trauma. Given the impact of childhood trauma on subjective cognitive functioning, screening for it is vital. While preventing childhood trauma is challenging, recent studies suggest that mitigating depressive symptoms can help preserve subjective cognitive functioning (Toyoshima et al., 2020).

Besides, a strong association was observed between childhood neglect and poor QoL in bipolar patients in the study, aligning with one study which reported that physical and emotional neglect might be a risk factor of MCS in euthymic bipolar patients (Serafini et al., 2016). Neglect could be as harmful as abuse in the long term but has received limited attention. The impact of neglect on QoL may be partially explained by socioeconomic position. Adolescents who born in the disadvantaged neighborhoods or living in low-income families were significantly more likely to experience childhood trauma than others (Walsh et al., 2019). Houtepen et al.’s (2020) have demonstrated that most of the childhood trauma types were associated with poor educational attainment and the strongest associations were seen for emotional neglect. Our results partly contrast with a previous study (Erten et al., 2014), which used SF-36 and found only the pain could be affected by childhood trauma in bipolar I disorder. This discrepancy may stem from their broader trauma group definition. Nonetheless, potential mechanisms linking childhood trauma to poor QoL include sensory processing disorders, depressive symptoms, low social support, and upsetting life events (Lin et al., 2018; Serafini et al., 2016). Furthermore, the role of interpersonal and personality vulnerabilities associated with childhood trauma warrants consideration. Childhood trauma is an established risk factor for the development of insecure attachment styles and borderline personality features (BPD), both of which are highly prevalent in BD and contribute to depression severity, resilience and functional impairment (Aydın et al., 2024; Citak and Erten, 2021; Knapen et al., 2025; Wrobel et al., 2022). Our finding revealed that High Neglect was linked to lower quality of life, potentially due to trauma-induced attachment insecurity (Wrobel et al., 2022). BD and BPD overlap in emotional dysregulation, with BPD linked to greater deficits in emotion recognition (Hestbaek et al., 2025). Emotional maltreatment in childhood is strongly linked to both disorders and can predict emotional blunting and cognitive problems (Hestbaek et al., 2025). Thus, within our sample, specific childhood trauma histories may contribute to functional impairments through mechanisms involving subtle BPD features or insecure attachment, further increasing daily psychosocial challenges. Future research should assess attachment and BPD symptoms to better understand their roles as mediators or moderators between childhood trauma and functional outcomes in BD.

### 4.1 Strength and limitations

Our study has several limitations. Firstly, retrospective surveys of childhood trauma may be influenced by recall bias and social expectations. Secondly, we cannot control for confounders induced by medication-specific factors, comorbidities, metabolic syndrome on cognition and QoL. Most patients received combined pharmacological treatments and were enrolled in studies of varying dosages, although the exact dosage of medicine was not precisely assessed. Lastly, the cross-sectional design limits causal inferences. Longitudinal studies are necessary to confirm and extend our findings. Furthermore, we did not evaluate borderline personality features or attachment styles, which are important psychosocial factors associated with both childhood trauma and functional outcomes in BD. Despite these limitations, the strengths of our study include a relatively large sample size (*N* = 275), use of healthy comparison group that have been better characterized in previous research. Furthermore, we illustrate for the first time that the associations between childhood trauma and subjective cognitive functioning in affective disorders.

## 5 Conclusion

In summary, the bipolar patients in the current study belonged to one of three latent profiles: i) HT, high levels of all types of trauma; ii) HN, high levels of emotional neglect and physical neglect; iii) LT, minimal trauma across all five types of the CTQ. Notably, only the HT profile was the risk factor of nearly all global and domain-specific cognitive functioning compared to LT, while the HN profile was specifically associated with poor quality of life and its physical and mental health subtypes. Both HT and HN profiles were linked to poor subjective cognitive functioning. Further research is crucial to elucidate the impact mechanism of trauma and neglect in BD, enhancing our understanding and guiding targeted interventions.

## Data Availability

The original contributions presented in the study are included in the article/Supplementary material, further inquiries can be directed to the corresponding author/s.
